# Intestinal obstruction caused by small bowel entrapment within a lumbar fracture: A case report

**DOI:** 10.1097/MD.0000000000031273

**Published:** 2022-10-21

**Authors:** Jin Suk Lee, Se Heon Kim, Jin Young Lee, Jin Bong Ye, Young Hoon Sul, Junepill Seok, Su Young Yoon, Hong Rye Kim, Jung Hee Choi, Yook Kim

**Affiliations:** a Trauma Surgery, Chungbuk National University Hospital, Cheongju, South Korea; b Trauma Surgery, College of Medicine, Chungbuk National University, Cheongju, South Korea; c Department of Thoracic and Cardiovascular Surgery, Chungbuk National University Hospital, Cheongju, South Korea; d Department of Neurosurgery, Chungbuk National University Hospital, Cheongju, South Korea; e Anesthesiology and Pain Medicine, Chungbuk National University Hospital, Cheongju, South Korea; f Department of Radiology, Chungbuk National University Hospital, Cheongju, South Korea.

**Keywords:** blunt trauma, bowel entrapment, vertebral fracture

## Abstract

Intestinal obstruction associated with traumatic vertebral fracture is extremely rare. We report a case of obstructive small bowel injury caused by entrapment of the small intestine at the fracture site of the 5^th^ lumbar vertebra due to trauma.

**Case presentation:**

A 55-year-old man fell from a height of 4 m and visited the emergency room of a local hospital with complain of back pain. During the examination, a 5^th^ lumbar vertebral body fracture and left psoas muscle hematoma were observed, and the patient was admitted to the neurosurgery department for conservative treatment. The patient received conservative treatment for 2 days, but new symptoms of intestinal obstruction and fever occurred. A neurosurgeon at the hospital suspected duodenal perforation and transferred the patient to the regional trauma center for treatment. Our medical staff reviewed the patient’s symptoms and imaging data and decided to perform an emergency operation because of small bowel entrapment in the 5^th^ lumbar vertebrae fracture and perforation of the small intestine. We found that the small bowel, approximately 160 cm below the ligament of Treitz, was incarcerated at the 5^th^ lumbar vertebral fracture site. After careful manual reduction of the entrapment of the small intestine, a small bowel resection of 25 cm, including the injury site, was performed with anastomosis.

**Conclusion:**

If symptoms of intestinal obstruction are observed in patients with traumatic spinal injury, medical staff must consider the exceedingly rare possibility of bowel entrapment.

## 1. Introduction

Intraperitoneal adhesions, hernias, and tumors are the main causes of mechanical bowel obstruction.^[1]^ Occasionally, mechanical bowel obstruction due to blunt trauma occurs^[2]^; however, mechanical obstructive bowel injury caused by a vertebral fracture or dislocation site is extremely rare.^[3,4]^ We report a rare case of obstructive small bowel injury caused by entrapment of the small intestine between a fracture site of the 5^th^ lumbar vertebra due to trauma.

## 2. Case presentation

Informed patient consent and ethical approval were obtained for the publication of this case report. A 55-year-old man with no underlying medical history visited the local emergency medical center because of back pain after falling from a height of 4 m. Examination revealed a fracture in the 5^th^ lumbar vertebral body and hematoma in the left psoas muscle. The patient was admitted to the neurosurgery department for conservative treatment. On the second day of hospitalization, the patient complained of abdominal distention, pain, a high fever. The attending physician performed abdominal pelvic computed tomography (CT) for evaluation. Retroperitoneal gas was observed on abdominal CT, and the medical staff suspected a traumatic duodenal perforation; therefore, the patient was referred to a regional trauma center.

**Figure 1.**
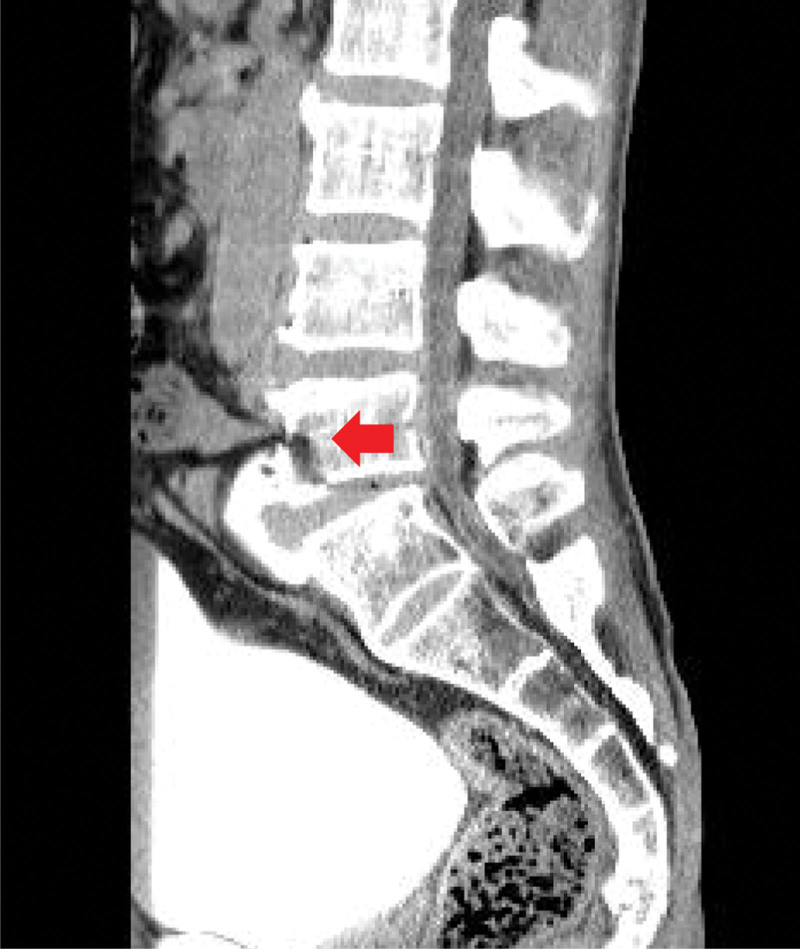
Bowel entrapment within a lumbar fracture.

**Figure 2.**
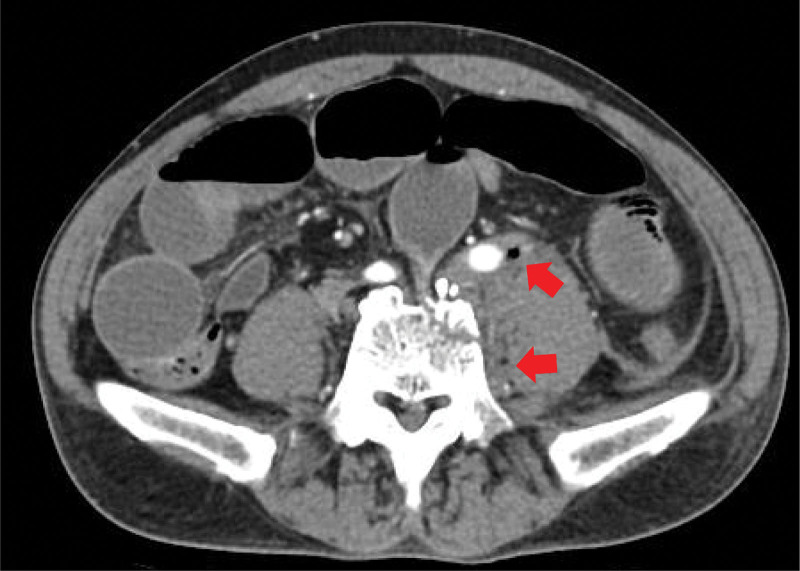
Air bobbles observed in the psoas muscle.

**Figure 3.**
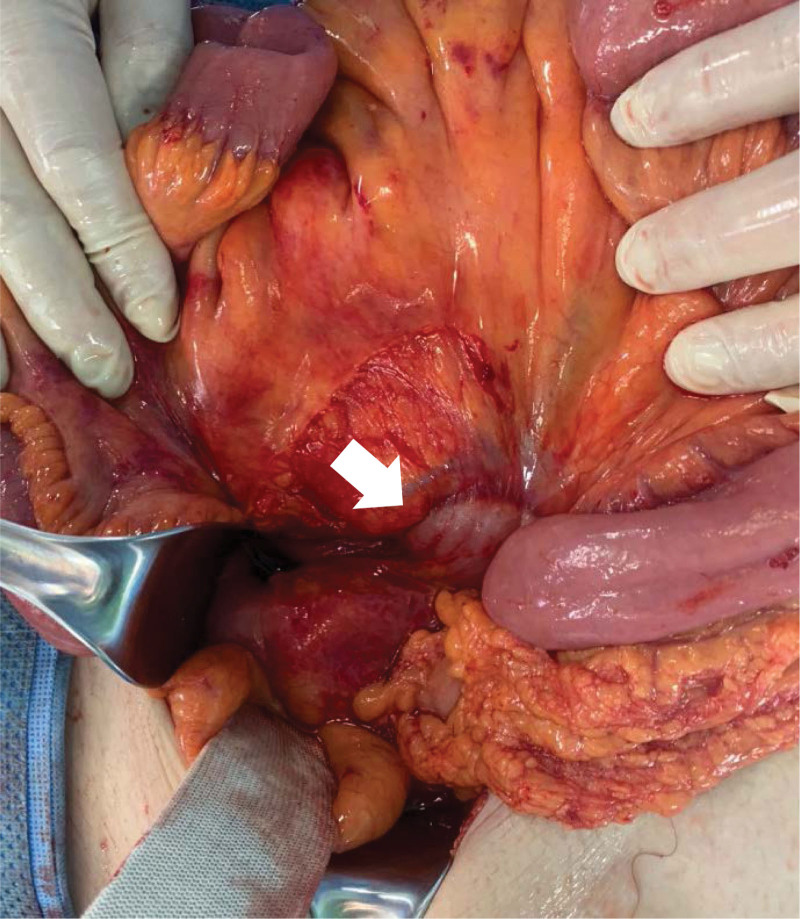
Small intestine was caught into lumbar vertebral body.

When the patient arrived at our trauma center, vital signs were as follows: systolic blood pressure, 80 mm Hg; heart rate, 112 beats/minute; respiration rate, 24 breaths/minute; body temperature 37.7°C; and oxygen saturation, 96%. The patient complained of lower back pain and abdominal distention. Our medical staff performed fluid resuscitation to stabilize the patient’s vital signs. The patient’s vital signs were as follows: systolic blood pressure, 114 mm Hg; heart rate, 115 beats/minute; respiration rate, 22 breaths/minute; body temperature, 38.2°C; and oxygen saturation, 96%. We reviewed the patient’s clinical symptoms and radiological findings and confirmed that the small intestine was pinched between the 5^th^ lumbar vertebral body fracture site (Fig. 1). In addition, several air bubbles were identified in the psoas muscle due to the perforation of the small bowel (Fig. 2). Therefore, an emergency surgery should be performed. A laparotomy was immediately performed, and exploration was performed along the dilated proximal small bowel. We found that the region approximately 160 cm below the ligament of Treitz passed through the retroperitoneum and mesentery injury and small bowel entrapment into the 5^th^ lumbar vertebral body fracture (Fig. 3). The distal area of the small intestine caught in the fracture area collapsed, and after careful reduction of the small bowel from the fracture site, perforation of the bowel was confirmed. We performed small bowel resection of the small intestine (approximately 25 cm in size), including the injured bowel, with an end-to-end anastomosis. To cover the exposed vertebral fracture area, we added a suture between the lacerated mesentery and the retroperitoneum. Subsequently, the accompanying vertebral fracture site was consulted by a neurosurgeon who decided to undergo conservative treatment without surgery. The patient’s condition improved, and he was discharged on the 10^th^ postoperative day without any complications.

## 3. Discussion

Most gastrointestinal injuries caused by blunt trauma are caused by an explosive increase in the intramural pressure of the intestine due to sudden pressure from a hard object outside the body or a hard structure within the body, such as the spine or pelvis.^[5]^ As in our case, traumatic vertebral fractures or dislocation-induced intestinal entrapment are extremely rare, and a study published in 2019 indicated that only 12 cases were reported from 1979 to 2016.^[4]^ It is not a direct injury to the intestine due to instantaneous force from trauma, but rather a severe hyperextension injury of the spine, which is induced by torn anterior longitudinal ligament and herniation of the intestine.^[6,7]^ This is due to obstruction and bowel strangulation caused by hernias may appear as symptoms. Similarly, esophageal injury may rarely occur because of hyperextension injury in cervical or thoracic spinal fractures.^[8,9]^

Uncommonly, blunt trauma can result in mesenteric injury, local bowel ischemia, or internal stenosis of the intestinal tract due to intestinal mucosal infarction, which can lead to mechanical obstruction.^[2,10]^ However, as in this case, there is a difference in the mechanism of mechanical obstruction caused by intestinal herniation due to traumatic vertebral fractures or dislocations. In cases of bowel obstruction due to intestinal stenosis, symptoms may appear delayed.^[10]^ However, in most cases, when the intestine is caught between the dislocated or fractured vertebrae, symptoms of obstruction are mainly observed at the early stage of trauma, and in severe cases, it is accompanied by bowel perforation.^[3,4,7,11]^ If bowel entrapment due to a fracture or dislocation of the spine is suspected, surgical treatment is necessary to manage the risk of complications or subsequent infection due to intestinal perforation.^[4]^

**Figure 4.**
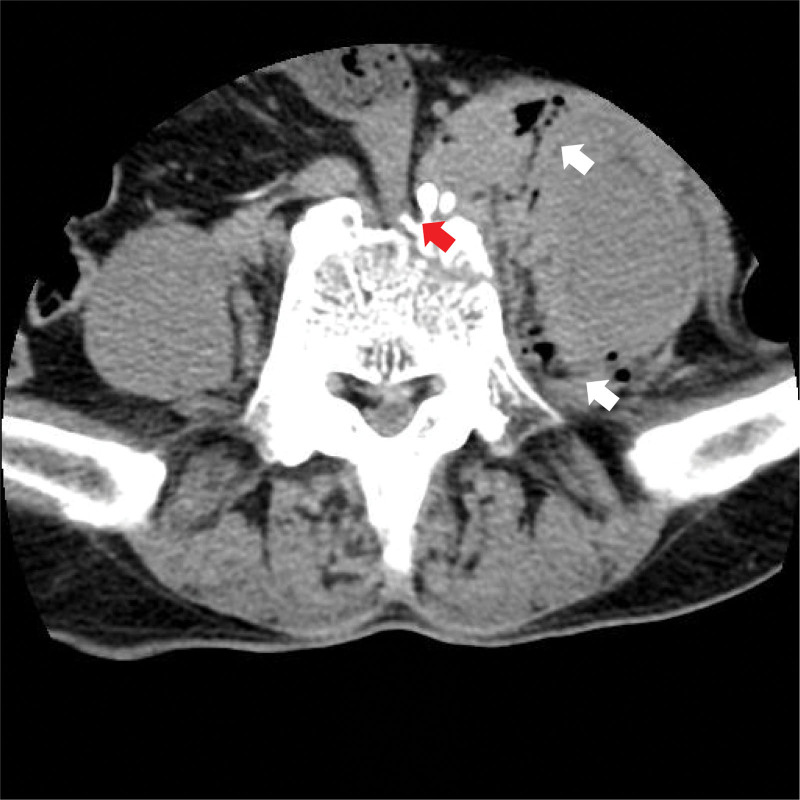
Several air bubbles are observed in the Psoas muscle, and the intestine is stuck in the lumbar vertebrae fracture.

Therefore, if symptoms of obstruction appear in the early stages of spinal trauma, careful differentiation from other causes is necessary. In patients with vertebral fractures due to trauma, bowel obstruction is commonly caused by paralytic ileus related to retroperitoneal hematoma.^[4]^ Paralytic ileus is caused by immobility due to spinal injury or the postoperative state, and opioid analgesics used for pain control also reduce gastrointestinal motility.^[12,13]^ As paralytic ileus and mechanical obstruction can be distinguished with a simple physical examination, they can be easily differentiated if the medical staff is suspected. However, because the occurrence of mechanical obstruction due to vertebral fracture is very rare,^[2]^ this possibility may not be considered. In our case, when the patient first complained of symptoms and visited the emergency room of a local secondary hospital, abdominal CT showed bowel dilatation and the small intestine stuck at the 5^th^ lumbar body fracture site (Fig. 4). Unfortunately, the medical staff could not diagnose the condition because they did not consider the possibility of mechanical obstruction caused by vertebral fracture.

## 4. Conclusion

Although bowel entrapment due to blunt trauma-induced spine fracture or dislocation is very rare, serious conditions, such as bowel strangulation or peritonitis, can occur. Therefore, if symptoms of intestinal obstruction are observed in patients with traumatic spinal injury, appropriate monitoring and physical examination should be performed to consider the exceedingly rare possibility of bowel entrapment. Above all, the suspicion of the medical staff is of utmost importance.

## Author contributions

Jin Suk Lee contributed to the acquisition of patient’s data and drafting of the manuscript; Se Heon Kim contributed to study conception and design and critical revision of the manuscript; All authors reviewed and approved the final submitted manuscript.

Conceptualization: Jin Suk Lee, Se Heon Kim.

Data curation: Jin Young Lee, Jin Bong Ye, Young Hoon Sul

Supervision: Se Heon Kim, Junepill Seok, Su young Yoon, Hong Rye Kim, Jung Hee Choi, Yook Kim.

Writing – original draft: Jin Suk Lee.

Writing – review & editing: Se Heon Kim.
